# Inheritance of Struggle: How Economic Inequality Fuels Depression Across Generations

**DOI:** 10.31083/AP53180

**Published:** 2026-04-22

**Authors:** Jaewon Lee, Jennifer Allen

**Affiliations:** ^1^Department of Social Welfare, Inha University, 22212 Incheon, Republic of Korea; ^2^School of Social Work, Michigan State University, East Lansing, MI 48824, USA

**Keywords:** depression, motherhood, young adulthood, intergenerational transmission, poverty, income

## Abstract

**Background:**

There have been few nationally representative studies on the association between intergenerational economic resources and mental health, particularly in the context of the mother-child relationship. The purpose of this study was to examine the association between maternal poverty and young adult children's depression and to test the mediating role of young adult children's income on the relationship.

**Methods:**

The data were collected from the National Longitudinal Survey of Youth 1979 (NLSY79) and the National Longitudinal Survey of Youth 79 for Children and Young Adults (NLSY79 CY). The two data sets were merged, 4224 pairs of mothers and their young adult children were selected, and a mediation model was conducted.

**Results:**

Mothers' poverty was significantly associated with their young adult children's income and their young adult children's depression. Young adult children's income was also significantly associated with their depression. Results indicated evidence of a mediation of the association between maternal poverty and young adult children's depression by young adult children's income.

**Conclusions:**

Recognizing mothers as independent agents of mobility transmission is necessary to further understand intergenerational mobility by considering the relationship between mothers and children. Anti-poverty interventions or programs targeted toward mothers should be developed to reduce mental health problems amongst their young adult children. Educational programs meant to increase economic resources should also focus on women with children in order to improve intergenerational economic mobility and their children's depression in young adulthood.

## Main Points

∙ The findings highlight the presence of intergenerational economic inequality, particularly in the context of maternal income, thus extending prior research that has predominantly focused on paternal influences.

∙ The mediating role of young adults’ income on the relationship between maternal poverty and young adults’ depression symptoms suggests that lower economic attainment among children of impoverished mothers partially explains the association.

∙ The results emphasize the need for targeted anti-poverty interventions and financial education programs for both low-income mothers and their children to promote upward economic mobility and reduce mental health disparities.

## 1. Introduction

Many individuals will experience a mental health problem in their lifetime, and depression is a mental health problem of particular global prevalence and concern [1, 2]. Given the abundance of mental health problems such as depression among young adults, their reluctance to seek mental health care, and the resulting disease burden, it is necessary to understand how to address the high risk of depression among young adults [3, 4].

Income or poverty status is important to consider in the context of young adults’ depression, as young adults tend to have lower incomes than older adults, and having a lower income is generally associated with a higher risk for depression [5, 6]. Not only young adults’ own income, but also the income of their parents, may affect their likelihood to experience depression [7]. The effects of poverty extend beyond the individual, as all household members suffer from its associated economic challenges [8]. Few studies, however, have investigated the association between the intergenerational transmission of poverty and mental health, particularly depression, across generations in the United States [7, 9, 10]. Additionally, few studies have examined this relationship between mothers and their young adult children specifically, and as women’s rates of labor force participation and their contribution to household incomes have increased, mothers’ income or poverty status may particularly influence their children’s mental health [11]. Although there have been numerous studies examining the relationship between economic status and mental health, this study seeks to address two gaps in the literature. First, there have been few nationally representative studies on the relationship between intergenerational economic resources and depression, particularly between mothers and their children. Second, potential mediators of this relationship have not been adequately explored.

## 2. Prevalence of Depression and Income Levels in Young Adulthood

Evidence suggests the presence of an inverse relationship between income and depression; this is especially salient for young adults in the USA, as they are at particularly high risk for depression, and they tend to have lower incomes than adults in older age groups [12, 13, 14, 15, 16, 17]. From 2021 to 2023, in a nationally representative USA survey, adolescents and adults aged 12–19 years and adults aged 20–39 years reported the highest prevalence of depression (19.2% and 16.6%, respectively) [6]. In general, the prevalence of depression often decreases across the lifespan [6, 13, 14]. In contrast, income tends to increase across the lifespan, at least until older ages when workers may expect to retire [15].

As young adults are more likely to report lower incomes and higher rates of depression compared to older adults, it is of particular interest to examine the relationship between the two in this age group. Regardless of age, people with incomes below or near the USA federal poverty line experience higher rates of depression than those with incomes above the USA federal poverty line [6]. Further, in a study that compared a random sample of adult women in the United Kingdom (regardless of depression status) to a sample of adult women diagnosed with depression, women with lower incomes exhibited higher rates of psychiatric disturbance (25% in the working-class income group vs. 5% in the high- and middle-class income groups) [18]. Moreover, in a sample of unmarried and married mothers, evidence of financial hardship over a two-year period was associated with a higher risk of onset of depression among unmarried mothers, as well as a higher risk of chronic depression among all mothers [19]. Similarly, in a sample of low-income, unmarried, Black mothers, maternal depression was positively associated with financial strain [20]. Last, when examining unhoused mothers and mothers residing in low-income housing, researchers found that these women were more likely to report lifetime or current depression than mothers with secure housing [21].

Two studies also examined this relationship in mixed-gender samples [6, 22]. Results of a survey administered to Black men and women during an economic recession showed an inverse relationship between depression and socioeconomic status (SES) and a positive relationship between depression and economic stressors [22]. Moreover, nationally representative USA data from 2021 to 2023 showed that 22.1% of adolescents and adults in households with family incomes under or near the USA federal poverty line reported depression, compared to 7.4% in households at or above 400% of the USA federal poverty line [6].

## 3. The Intergenerational Transmission of Economic Resources and Depression

Researchers have theorized multiple pathways by which poverty in childhood may affect depression in adulthood. There is neurobiological and genetic evidence to suggest that stressors associated with experiencing childhood poverty can affect the brain structure in the corticolimbic system, a region which has been connected to psychopathology [23]. According to researchers attempting to outline an ecological neuroscience model in the context of developmental neuroscience, there are two ways that childhood poverty affects the developing brain: due to fewer resources owing to the experience of poverty, as well as increased stressors associated with poverty [23]. Further, researchers have found that stress related to childhood poverty can lead to changes in genes in pathways related to stress and inflammation that are associated with mental health issues, including (but not limited to) depression [24]. Children exposed to stressors experience a higher allostatic load, which may affect the Hypothalamic–Pituitary–Adrenal (HPA) axis’ ability to prevent the accumulation of excess cortisol, leading to higher hair cortisol concentrations in children exposed to stressors, such as children exposed to childhood poverty [24]. Research has shown that higher hair cortisol concentrations are associated with several mental health issues across the lifespan, including depression in adulthood [24]. Moreover, researchers have proposed that childhood poverty is associated with greater parental distress, as well as with negative effects on the parent-child relationship and on parenting style, all of which can mediate the relationship between childhood poverty and depression in adulthood [24, 25, 26]. Through the lens of attachment theory, the mother-child relationship is of particular interest when examining mental health in young adulthood, because research has found that individuals with secure attachments to a primary caregiver (often the mother) are at lower risk for mental health problems across the lifespan [27, 28, 29]. Individuals with insecure attachment styles (e.g., ambivalent, disorganized, resistant, or avoidant) are more likely to be depressed in childhood and adolescence than those with a secure attachment style [28, 30]. Examining mothers’ poverty is also of interest because research has found that insecurely attached children who are raised in higher risk environment, such as in poverty, show more symptoms of depression from childhood through adolescence than do securely attached children [28].

Evidence supports the idea that adults who grow up in poverty are at higher risk for depression in young adulthood and beyond, as compared to adults who did not grow up in poverty [7, 9, 10]. In one longitudinal study, poverty at age 14 years positively predicted anxiety and depression at ages 14 and 21 years, and participants who experienced poverty more frequently from infancy through age 21 years had higher rates of anxiety and depression [7]. In another retrospective study of adults aged 50 years at the time of data collection, childhood poverty was associated with current depression [10]. Further, in a cohort study that included people born between 1984 and 1988, adults who reported household public assistance use in childhood or who reported residential instability in childhood were more likely to have a clinical diagnosis of depression in adulthood, compared to those who reported neither [9]. Therefore, as evidence suggests an inverse relationship between childhood poverty and depression in adulthood, it is of interest for researchers to examine additional pathways between these two variables, in order to better understand the relationship and to identify more points of potential intervention to reduce the likelihood of depression in adulthood among those who experienced poverty as a child.

## 4. The Current Study

The purpose of this study is to examine the relationship between mothers’ poverty, their children’s income and depression in young adulthood. Young adults tend to have higher depression and lower income than older adults [4, 15]. Adults with lower incomes or who are living at or near the federal poverty line are more likely to report depression than adults with higher incomes [6]. Moreover, longitudinal studies have shown that adults who grew up in poverty were more likely to experience depression in adulthood than those who had not [7, 9, 10]. It is of interest to examine young adults’ income and depression in the context of their mothers’ poverty because the association between the mother-child relationship and the child’s depression across the lifespan may be affected by growing up in poverty [28]. For this study, our specific research questions are as follows: (1) Is there an association between mothers’ poverty and their young adult children’s income? (2) Is there an association between mothers’ poverty and their young adult children’s depression? and (3) Does young adult children’s income mediate the association between their mothers’ poverty status and their own risk for depression?

## 5. Methods

### 5.1 Data and Sample

This study utilized two secondary nationally representative data sets: the National Longitudinal Survey of Youth 1979 (NLSY79) and the National Longitudinal Survey of Youth 79 for Children and Young Adults (NLSY79 CY). Each dataset is nationally representative and managed by the USA Department of Labor. The sample of the NLSY79 included 12,686 individuals who were between the ages of 14 and 22 when data collection began in 1979. Participants were interviewed each year from 1979 to 1994, and biennially thereafter. The USA Department of Labor collected the NLSY79 data from 1979 to 2012 and the NLSY79 CY from 1986 to 2012. Both datasets are based on USA nationally representative samples. Information about the labor market and a variety of other dimensions was collected.

The current study focuses on participants’ economic status and resources as well as mental health. The NLSY79 provides mothers’ information and the NLSY79 CY provides their young adult children’s information. The young adult children are the biological children of the mothers in the NLSY79. In both data sets, the latest wave, collected in 2012, was used to pair mothers and children. The NLSY79 and NLSY79 CY were merged by using the mother and child’s identification number, and mothers were then matched with their children. Children who were not interviewed or declined to provide information about their depression were excluded. A total of 4224 pairs were selected for the final sample. Furthermore, since this study utilized publicly available secondary data, it contains no identifiable characteristics of the sample.

### 5.2 Measures

#### 5.2.1 Mental Health

In this study, young adult children’s mental health refers to levels of depression, measured via the Center for Epidemiologic Studies Depression Scale (CES-D). The CES-D includes 11 items based on a four-point Likert-type scale with responses ranging from 0 “rarely or none of the time (<1 day)” to 3 “most or all of the time (5–7 days)”. One item, measuring happiness, was reverse-coded before analysis. Scores were summed with higher scores indicating greater risk of depression (Mean = 5.10, SD = 5.03; Range = 0–32). The CES-D scale items loaded onto a single factor, indicating good internal consistency (Cronbach’s alpha = 0.80). The scale also shows correlations with other established measures of depression [31, 32]. Further, the shortened 11-item CES-D scale has demonstrated reasonable construct validity and utility in capturing the presence and severity of depressive symptom [33].

#### 5.2.2 Maternal Poverty

Poverty among mothers refers to where their annual income fell in the context of the USA federal poverty line. This variable was already computed and provided by the NLSY79. The answers were classified into two categories: Individuals whose incomes fell above the poverty line (coded = 0) and those whose incomes fell below the poverty line (coded = 1).

#### 5.2.3 Young Adult Children’s Income

Income consists of wages, salary, commissions, and/or job-related travel before taxes. The highest incomes were top-coded, in that those ranked in the highest percent of total income were excluded due to the possibility of recognition. This variable was then recoded by dividing by 10,000, with higher values indicating higher income (Mean = 1.49, SD = 2.17; Range = 0–15.32).

#### 5.2.4 Control Variables

Demographic characteristics and socioeconomic status were included as control variables. Age, marital status, and education were controlled for mothers and their young adult children, and children’s gender and race/ethnicity were also included.

### 5.3 Mediation Analysis

**Fig. 1.**
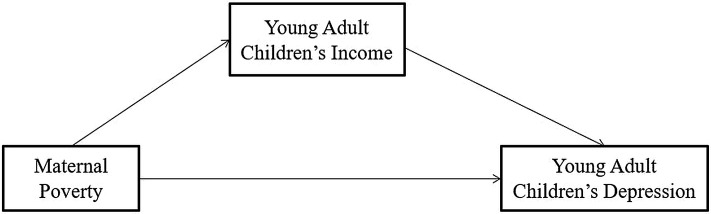
**Path diagram of the mediator model of young adult children’s income on maternal poverty and young adult children’s depression**.

A series of procedures suggested by Baron and Kenny [34] were conducted to test if the relationship between maternal economic status and their young adult children’s depression is mediated by their young adult children’s income. According to Baron and Kenny, the relationships among the independent variable, mediator, and dependent variable must satisfy the following three conditions: (1) the independent variable (maternal economic status: poverty) must influence the mediator (young adult children’s economic resources: income); (2) the independent variable must influence the dependent variable (young adult children’s depression); and (3) the mediator must influence the dependent variable [34]. A Sobel test will be conducted to test whether the mediation model is significant or not. Multiple mediation analyses were conducted to answer the research questions using the Statistical Package for the Social Sciences (SPSS) 22.0 (IBM Corp., Armonk, NY, USA). The derived mediation model is shown in Fig. 1.

## 6. Results

### 6.1 Descriptive Results

Descriptive statistics are in Table 1. For young adult children, the average depression score was 5.10 and the average income was $ 14,881. 46.6% were non-Hispanic White, 32.6% African American and 20.8% Hispanic. Nearly 51% were female, and their average age was 25 years old. About 15% were married, and less than 20% had received higher education. Among the mothers, about 22% were in poverty. Their average age was about 51 years old, about 54% were married, and just over one-third had received higher education.

**Table 1.** **Descriptive statistics for variables included in the study**.

Variable			Total (n = 4224)
			% or mean (SD)
Young adult child			
	Depression		5.10 (5.03)
	Income		1.49 (2.17)
	Demographics		
		Race (non-Hispanic White)	46.6%
		Gender (female)	50.9%
		Age	25.08 (5.93)
		Higher education	16.8%
		Marriage	15.1%
Mother			
	Poverty		21.6%
	Demographics		
		Age	50.59 (2.23)
		Higher education	33.4%
		Marriage	53.9%

Notes. The real values of income should be multiplied by 10,000.

### 6.2 Results of Mediation Model

As shown in Table 2, the first condition, that the independent variable must influence mediator, is confirmed as maternal economic status (poverty) was significantly associated with young adult children’s economic resources (income) (β = –0.47, *p* < 0.001), such that maternal poverty negatively influenced young adult children’s income. This indicates inequality in the intergenerational economic mobility between mothers and their young adult children (Research question 1). The second condition, that the relationship between the independent variable and dependent variable is significant, was also met. Table 3 shows that maternal poverty was significantly, positively associated with their young adult children’s depression (β = 0.83, *p* < 0.001). Young adult children were more likely to experience depression if their mother was in poverty (Research question 2).

**Table 2.** **Regression analysis of factors influencing young adult children’s income**.

Variables			Income	
(Constant)			–3.78 (0.77)	
Young adult child				
	Race			
		African American	–0.14 (0.08)	
		Hispanic	–0.06 (0.09)	
	Gender (female)		–0.49 (0.07)	^*⁣**^
	Age		0.15 (0.01)	^*⁣**^
	Higher education		1.13 (0.09)	^*⁣**^
	Marriage		0.67 (0.10)	^*⁣**^
Mother				
	Age		0.03 (0.02)	^+^
	Higher education		0.05 (0.08)	
	Marriage		0.02 (0.07)	
	Poverty		–0.47 (0.09)	^*⁣**^

Note. ^+^*p* < 0.10. ^*⁣**^p < 0.001.

**Table 3.** **Regression analysis of factors influencing young adult children’s depression**.

Variables			Depression	
(Constant)			4.21 (2.01)	
Young adult child				
	Race			
		African American	0.01 (0.22)	
		Hispanic	–0.43 (0.26)	^+^
	Gender (female)		0.87 (0.17)	^*⁣**^
	Age		0.10 (0.02)	^*⁣**^
	Higher education		–1.40 (0.25)	^*⁣**^
	Marriage		–1.70 (0.28)	^*⁣**^
Mother				
	Age		–0.02 (0.04)	
	Higher education		–0.14 (0.20)	
	Marriage		–0.45 (0.19)	^*^
	Poverty		0.83 (0.24)	^*⁣**^

Note. ^+^*p* < 0.10. ^*^p < 0.05. ^*⁣**^p < 0.001.

Table 4 shows that the third condition, that the mediator is related to dependent variable, was also met. Young adult children’s income was negatively associated with their depression (β = –0.24, *p* < 0.001). This indicates a significant mediation of the relationship between maternal poverty and their young adult children’s depression by the young adult children’s income (Research question 3). A Sobel test confirms that the mediation model is statistically significant (Z = 3.53; *p* < 0.001).

**Table 4.** **Mediation effect of young adult children’s income on maternal poverty-young adult children’s depression unstandardized coefficients (standard error)**.

Variables			Depression	
(Constant)			2.44 (2.09)	
Young adult child				
	Race			
		African American	0.05 (0.23)	
		Hispanic	–0.51 (0.24)	^*^
	Gender (female)		0.80 (0.18)	^*⁣**^
	Age		0.14 (0.02)	^*⁣**^
	Higher education		–1.03 (0.26)	^*⁣**^
	Marriage		–1.65 (0.29)	^*⁣**^
Mother				
	Age		–0.01 (0.04)	
	Higher education		0.07 (0.20)	
	Marriage		–0.49 (0.20)	^*^
	Poverty		0.77 (0.25)	^**^
Mediator				
	Young adult children’s income		–0.24 (0.05)	^*⁣**^

Note. ^*^p < 0.05. ^**^p < 0.01. ^*⁣**^p < 0.001.

## 7. Discussion

This study explored the mediating role of young adults’ income on the association between their mothers’ poverty and their own depression via a USA nationally-representative sample of mother-child pairs. Our findings demonstrated that maternal poverty was positively associated with their young adult children’s depression. The mediation model further indicated that young adult children’s income mediated the association linking their mothers’ poverty and their depression.

First, our findings showed that poverty among mothers was negatively related to their young adult children’s income. This finding aligns with previous research indicating evidence of inequality in intergenerational mobility [7, 9, 10]. Given that more research has addressed the intergenerational mobility between fathers and children [35, 36], our findings extend the work of previous studies by showing the mobility across generations of mothers and children. As more women participate in the labor force and contribute to the economy [37, 38], their economic influence on households should be considered to understand children’s economic resources. As attachment theory shows, the close relationship between mothers and children continues into adulthood [11, 39]. Therefore, maternal influences at an economic level should be considered to understand children’s economic resources in young adulthood. If mothers have fewer economic resources or assets, their children ultimately may have lower educational attainment, leading them to work at lower-paying jobs. Therefore, anti-poverty programs, such as financial assistance programs and educational programs for children from low-income households, are critical to reduce inequitable economic mobility between mothers and children.

Affirming findings from previous research [40, 41], this study also shows that poverty was associated with depression. However, compared to other studies, this study demonstrates that mothers’ poverty status is related to increased prevalence of depression in their young adult children. Based on attachment theory, adults, particularly with secure attachments, may maintain a strong cohesion with their mothers [11, 39, 42]. Evidence suggests that secure attachments are associated with a decreased risk of mental health issues, including depression, across the lifespan, and that living in poverty (or another high-risk environment) may exacerbate the negative effect of an insecure attachment on psychopathology [28, 29, 30]. As a result, the mothers’ financial burdens affect their children, potentially resulting in their children experiencing similar financial problems and poor mental health. Thus, mental health issues, such as depression, may more frequently occur in young adult children who grew up in poverty. Therefore, poverty prevention interventions or programs aimed at alleviating financial burdens among mothers in poverty, and mental health prevention programs for children in low-income households, should be developed to alleviate generational income inequality and future mental health problems among their children.

Moreover, the significant mediation effect of young adult children’s income in the association between maternal economic status and young adult children’s depression suggests that lower maternal economic status is linked to poorer economic resources among young adults, which in turn places them at greater risk for depression. Therefore, intergenerational economic mobility is one important issue to consider when working to reduce depression. In particular, this study reveals that the inequitable income mobility across generations between mothers and children should be given more attention when considering young adults’ depression. Thus, anti-poverty and financial education programs targeted toward mothers as well as financial assistance for young adults who grew up in poverty may be important to improve intergenerational economic mobility and young adults’ depression.

This study’s findings may be considered in light of its limitations. As this study was conducted based on cross-sectional data, the mediation model is limited when examining the relationships across generations. A longitudinal approach may be beneficial to further understand the relationship and direction of effects. Second, due to a correlation issue, this study measured economic resources by poverty and income. Including additional economic factors in future studies may be helpful to further identify mediational pathways. Third, this study only focused on the effects of maternal economic status on young adults’ depression. The presented mediational pathway needs to be further examined in different age groups, such as middle aged and older adult children.

## 8. Conclusions

There have been few nationally representative studies on the association between intergenerational economic resources and mental health, particularly when regarding the mother-child relationship. A large body of previous research has primarily addressed the effects of family poverty on psychological health, rather than examining maternal poverty specifically. By focusing solely on maternal poverty, this study highlights the unique influence of mothers on their children’s depression in young adulthood, addressing a research gap that has been overlooked in previous studies. Our findings expand upon previous research by investigating mobility across generations between mothers and children. Unequal income mobility across generations, particularly between mothers and children, should be more considered to prevent and reduce depression among young adults. Based on this discussion, we suggest that future studies focus on the intergenerational transmission between mothers and their children using longitudinal data to gain a deeper understanding of depression in young adulthood. In addition, to strengthen causal inference, we proposed measuring maternal poverty during the child’s adolescence, as this enhances the robustness of the analysis. To assess economic status, future studies should consider incorporating additional indicators.

## Availability of Data and Materials

The data is available at the official website of the National Longitudinal Surveys https://www.nlsinfo.org/.
